# SARS-CoV-2 Infection of Airway Epithelium Triggers Pulmonary Endothelial Cell Activation and Senescence Associated with Type I IFN Production

**DOI:** 10.3390/cells11182912

**Published:** 2022-09-17

**Authors:** Veronica Bordoni, Davide Mariotti, Giulia Matusali, Francesca Colavita, Eleonora Cimini, Giuseppe Ippolito, Chiara Agrati

**Affiliations:** 1 Cellular Immunology Laboratory, National Institute for Infectious Diseases “L. Spallanzani” IRCCS, Via Portuense 292, 00149 Rome, Italy; 2 Laboratory of Virology, National Institute for Infectious Diseases “L. Spallanzani” IRCCS, Via Portuense 292, 00149 Rome, Italy; 3 General Directorate for Research and Health Innovation, Italian Ministry of Health, 00144 Rome, Italy

**Keywords:** COVID-19, endothelial cells, airway epithelium, senescence, IFN-β

## Abstract

Airway epithelial cells represent the main target of SARS-CoV-2 replication but several pieces of evidence suggest that endothelial cells (ECs), lining pulmonary blood vessels, are key players in lung injury in COVID-19 patients. Although in vivo evidence of SARS-CoV-2 affecting the vascular endothelium exists, in vitro data are limited. In the present study, we set up an organotypic model to dissect the crosstalk between airway epithelium and pulmonary endothelial cells during SARS-CoV-2 infection. We showed that SARS-CoV-2 infected airway epithelium triggers the induction of endothelial adhesion molecules in ECs, suggesting a bystander effect of dangerous soluble signals from the infected epithelium. The endothelial activation was correlated with inflammatory cytokines (IL-1β, IL-6, IL-8) and with the viral replication in the airway epithelium. Interestingly, SARS-CoV-2 infection determined a modulation of endothelial p21, which could be partially reversed by inhibiting IFN-β production from ECs when co-cultured with HAE. Altogether, we demonstrated that SARS-CoV-2 infected epithelium triggers activation/senescence processes in ECs involving type I IFN-β production, suggesting possible antiviral/damage mechanisms occurring in the endothelium.

## 1. Introduction

Airway epithelial cells represent the main target of SARS-CoV-2 replication, but several evidences suggest that endothelial cells (ECs) can be also infected [1,2,3].

In the initial stage of severe COVID-19 patients, SARS-CoV-2 infection causes acute lung injury, associated with massive inflammatory cytokines released by immune cells, bronchial epithelial cells, and alveolar cells. The Spike protein of SARS-CoV-2 can cause direct damage to endothelial cells, and induce the angiotensin-converting enzyme 2 (ACE2) downregulation, which may further worsen endothelial dysfunction [4,5]. SARS-CoV-2 infection and several cytokines, such as IL-6, IL-1β, and TNFα, participate in endothelial activation and dysfunction, leading to vascular inflammation, an increase in permeability, and vasculitis. Endothelial cells contribute to cytokine production that, in turn, augments platelet production and activation [6,7]. Inflammation, edema, and microthrombus work together to cause ARDS.

The ECs activation/damage in SARS-CoV-2 infected patients is well demonstrated, but the direct role of viral infection or the involvement of a bystander process remains to be investigated. Moreover, cellular senescence is now considered an important driving process that may participate in the functional tissue impairment observed during stress or infection conditions [8,9,10].

The cell adhesion molecules considered as early markers of endothelial activation and dysfunction, e.g., selectins (E-, P- and L-selectin), ICAM-1, and VCAM-1 [11], are found elevated in plasma samples of COVID-19 patients [12,13], and may be associated to vascular damage [14]. VCAM-1 is a monocyte endothelial ligand that mediates monocytes’ recruitment from the bloodstream to sites of infection and injury [15]. ICAM-1, expressed on the surface of ECs, is upregulated in lesions and supports leukocyte recruitment and adhesion [13,16]. E-selectin (CD62E) is produced solely by ECs after their activation by pro-inflammatory factors, mediates leukocyte adherence to the activated EC surface, and acts as a chemotactic molecule for phagocytic cells [17].

Cellular senescence may contribute to the host response to viral infections: the activation of senescence-associated pathways can lead to the secretion of inflammatory mediators, which may either promote host defense or exacerbate immune pathology during viral infections [18]. Cellular senescence is regulated by replicative and stress-related signals with activation of p53 and p16INK4a, leading to p21 activation and cell cycle arrest. Moreover, increased p21 expression in epithelial cells as well as in endothelial cells was detected in COVID-19 lung autopsy case series [19].

Altogether, these findings suggest that endothelial cell activation and damage play a central role in the pathogenesis of COVID-19 associated with acute respiratory disease syndrome, pulmonary edema, diffuse coagulopathy, and multiple organ failure [20,21].

Although there is evidence of SARS-CoV-2 affecting in vivo the vascular endothelium, in vitro data on the mechanisms involved in such events are limited [1,15,22]. Moreover, the interplay between pulmonary epithelial and endothelial cells upon SARS-CoV-2 infection is still largely unexplored as well as the dissection of the molecular processes (viral and host-mediated) in triggering endothelial damage. In a recent paper, we took advantage of using a reconstituted organotypic human airway epithelium (HAE) to analyze the interplay between SARS-CoV-2 infected epithelium and lympho-monocytes, highlighting as immune cells strongly affected the inflammatory profile induced by SARS-CoV-2 infected epithelium [23].

The aim of this study was to evaluate the impact of SARS-CoV-2 infection on endothelial activation/damage in a co-culture model of organotypic HAE and pulmonary ECs. Specifically, we investigated: (i) the ability of EC in sustaining SARS-CoV-2 replication (iEC vs. EC); (ii) the impact of HAE on EC-viral replication in co-culture (iEC vs. iEC/HAE); (iii) the by-stander effect of iHAE on EC damage/senescence (iHAE/EC vs. HAE/iEC).

## 2. Materials and Methods

### 2.1. Human Airway Epithelium (HAE) Culture

Human Bronchial Epithelial Cells (HBEpCs, from PromoCell, Heidelberg, Germany) were cultured in Bronchial/Tracheal Epithelial Cell Growth Medium (Cell Applications, San Diego, CA, USA), supplemented with 2 mM L-glutamine, 50 U/mL penicillin, and 50 μg/mL streptomycin, in a humidified atmosphere (5% CO_2_) at 37 °C, to allow HBEpCs to expand. Once the cells were well expanded, they were passed in PneumaCult™Ex Plus Medium (STEMCELL, Vancouver, BC, Canada), supplemented with 2 mM L-glutamine, 50 U/mL penicillin, and 50 μg/mL streptomycin, in a humidified atmosphere (5% CO_2_) at 37 °C. Once confluence was reached, cells were detached using the Animal Component-Free (ACF) Cell Dissociation Kit (STEMCELL, Vancouver, BC, Canada) and seeded in 12 mm transwell inserts (STEMCELL, Vancouver, BC, Canada) at the density of 1.1 × 10^5^ cells/mL with the same medium at the apical (0.5 mL) and basal (1 mL) sides, until confluence was reached. Afterward, the medium was cleared from both apical and basal sides, adding PneumaCult™-ALI-S Medium (STEMCELL, Vancouver, BC, Canada) only in the lower chamber, leaving the apical chamber in contact with the air and starting the differentiation. The medium was changed every two days until complete differentiation in Human Airway Epithelium (HAE), observable from mucus production, which can be removed using 0.5 mL of Phosphate buffered saline (PBS) 1× (from Corning Incorporated, Corning, New York, NY, USA).

### 2.2. Human Pulmonary Microvascular Endothelial Cells (ECs) Culture

Human Pulmonary Microvascular Endothelial Cells (ECs, PromoCell Heidelberg, Germany) were cultured in Endothelial Cell Growth Medium MV 2 (PromoCell, Heidelberg, Germany), supplemented with 50 U/mL penicillin, and 50 μg/mL streptomycin, in a humidified atmosphere (5% CO_2_) at 37 °C, to allow ECs to expand. Once confluence was reached, cells were detached using the Animal Component-Free (ACF) Cell Dissociation Kit (STEMCELL Vancouver, BC, Canada) and seeded or on a round polylysinate 12 mm slide placed in a 12 well plate or directly on the bottom of a 12 well plate (Corning Incorporated, New York, NY, USA) at a density of 1.1 × 10^5^ cells/mL with 1 mL of medium, until confluence was reached.

### 2.3. SARS-CoV-2 Infection and HAE-EC Co-Culture

HAE or ECs cells were infected separately with SARS-CoV-2, (Human 2019-nCoV strain 2019-nCoV/Italy-INMI1, clade V; RefSKU: 008V-03893 European Virus Archive—GLOB-AL, GISAID: BetaCoV/Italy/INMI1-isl/2020: EPI_ ISL_410545) in MEM at a multiplicity of infection (M.O.I.) of 0.1. Viral inoculum or medium only (not infected) was applied on the cells for 1 h and 30′ at 37 °C and 5% CO_2_. Viral inoculum was then removed and cells were washed with 0.3 mL of 1×PBS two times. Next, 1 mL of Endothelial Cell Growth Medium MV 2 was added to each well containing ECs; HAE were then co-cultured with ECs (on transwell inserts), and maintained in a humidified atmosphere (5% CO_2_) at 37 °C for two or six days. At the end of the culture, we collected HAE, ECs, and supernatants. The viability of cells was verified by Trypan blue exclusion. 

### 2.4. H-151 Treatment

H-151 (InvivoGen, San Diego, CA, USA) was reconstituted in Dimethyl Sulfoxide (vehicle) according to the manufacturer’s instructions and used at the concentration of 1 µM after dilution in Endothelial Cell Growth Medium. After removing SARS-CoV-2’s inoculum in iHAE, H-151 (or vehicle) was diluted in Endothelial Cell Growth Medium MV 2 and added to EC wells. After two days of coculture iHAE-EC, we collected HAE, ECs, and basal/apical supernatants. 

### 2.5. Real-Time RT-PCR

Total RNA was extracted from HAE and ECs using Direct-zol™ RNA MicroPrep KIT (from Zymo Research Corp., Irvine, CA, USA) according to the manufacturer’s instructions. For RT-PCR analysis, single-stranded cDNA was obtained by reverse transcription of one μg of total RNA using AMV-reverse transcriptase (from Promega Corporation, Madison, WI, USA). ICAM-1, VCAM-1, E-selectin, IFNβ, p21, and p53 mRNA levels were analyzed by quantitative PCR using the following primers: ICAM-1 F: 5′-CTCCAATGTGCCAGGCTTG-3′ R: 5′-CAGTGGGAAAGTGCCATCCT-3′; VCAM-1 F: 5′-TTCCCTAGAGATCCAGAAATCGAG-3′ R: 5′-CTTGCAGCTTACAGTGACAGAGC-3′; E-selectin F: 5′-TGAAGCTCCCACTGAGTCCAA-3′ R: 5′-GGTGCTAATGTCAGGAGGGAGA-3′; IFNβ F: 5′-CTTGGATTCCTACAAAGAAGCAGC-3′ R: 5′-TCCTCCTTCTGGAACTGCTGCA-3′; p21 F: 5′-TCAAAGGCCCGCTCTACATCTTCT-3′ R: 5′-TAGGAACCTCTCATTCAACCGCCT-3′; p53 F: 5′-GCCGTCCCAAGCAATGGATGATTT-3′ R: 5′-TCTGGCATTCTGGGAGCTTCATCT-3′. qPCR was performed with FastStart Essential DNA Green Master (from Roche, Basel, Switzerland) according to the manufacturer’s instructions. The expression levels were normalized to the GAPDH level (GAPDH F: 5′-GGTGGTCTCCTCTGACTTCAACA-3′, R: 5′-GTGGTCGTTGAGGGCAATG-3′) using the equation “2^−ΔCt^”. All qPCR reactions were performed in a Corbett 212 Rotor-gene6000 (Sydney, New South Wales, Australia) Real-Time PCR System.

### 2.6. Immunofluorescence

HAE on transwells and ECs on round polylysinate slides, after two days from infection, were stained as previously described [18]. Briefly, cells were fixed and transwells were cut and laid down, together with slides, in a new 24-cell culture well plate with the side on which cells are located face-up. Then, transwells and slides were blocked with Blocking Buffer for one hour at RT. Later, the blocking buffer was removed and primary antibodies (anti-ZO-1 from Invitrogen (Waltham, MA, USA); anti-SARS-CoV-2 spike antibody from GeneTex (Irvine, CA, USA) diluted 1:300 were added to each transwell/slide and incubated overnight at 4 °C in the dark. The following day primary antibodies were removed and 0.45 mL of secondary antibodies (anti-Mouse IgG1 AF488 and anti-Rabbit IgG Alexa-647 from Sigma Aldrich (St. Louis, MO, USA) diluted 1:1000 were added to each transwell/slide and incubated for two hours at RT in the dark. Afterward, cells were mounted side up on a slide using one drop of Mounting with DAPI. In the end, images were acquired using a Leica THUNDER 3D Live Cell Imaging System (Wetzlar, Germany) at 63× magnification. 

### 2.7. Cytokines Quantification

Soluble inflammatory cytokines (sIL-1β, sIL-6, sIL-8) and IFN-β were quantified in HAE-EC culture supernatants using a customized automatic ELISA (ELLA from Protein Simple, San Jose, CA, USA).

### 2.8. Viral Quantification

Viral quantification has been performed on HAE and EC. Briefly, cells were collected and total RNA was extracted from HAE and ECs using Direct-zol™ RNA MicroPrep KIT (from Zymo Research Corp., Irvine, CA, USA) according to the manufacturer’s instructions. Real time RT-PCR was performed on 40 ng of cell-associated RNA using the RealStar^®^ SARS-CoV-2 RT-PCR Kit RUO (Altona Diagnostics, Hamburg, Germany), which amplifies the E- and S- viral genes.

### 2.9. Statistical Analysis

Quantitative variables were compared with nonparametric Wilcoxon and Mann-Whitney tests. A *p*-value lower than 0.05 was considered statistically significant. Statistical analyses were performed using GraphPad Prism v8.0 (GraphPad Software, Inc., San Diego, CA). To visualize a correlation matrix in R we used the corrplot function and generate a Heatmap object using correlation coefficients (computed using the Spearman) as input to the Heatmap. The heatmap was produced with the R package heatmap3. More details of the statistical analysis are included in Figure legends.

## 3. Results

### 3.1. Co-Culture of SARS-COV-2 Infected Airway Epithelium with Microvascular Pulmonary Endothelial Cells: The Experimental Model

**Figure 1:**
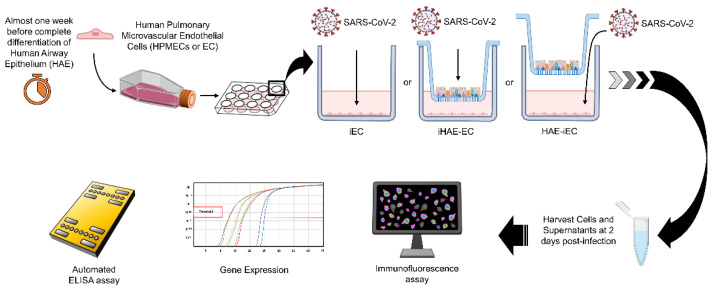
Experimental model and design. Almost one week before the complete differentiation of Human Airway Epithelium (HAE), Human Pulmonary Microvascular Endothelial Cells (ECs) were thawed and expanded. HAE were then co-cultured with ECs (HAE-EC). Afterward, three different culture conditions were performed: (i) ECs were infected and cultured alone (iEC); (ii) ECs were infected and cultured with HAE (HAE-iEC) and (iii) uninfected ECs were cultured with infected HAE (iHAE-EC). Samples from infected cultures (supernatants and cells) were collected for inflammatory cytokines quantification, gene expression, and immunofluorescence analysis at 2-days post-infection.

To set up a co-culture model, we cultured primary human bronchial epithelial cells at an air-liquid interface (human airway epithelium, HAE) as previously described [23] with primary human microvascular pulmonary endothelial cells (ECs). To dissect the crosstalk between epithelium and endothelium, we challenged separately the ECs (infected ECs, iEC), or the apical surface of the epithelium (infected HAE, iHAE) with SARS-CoV-2. Soon after the removal of viral inoculum, ECs were cultured alone (iEC) or in co-culture with HAE (HAE-iEC or iHAE-EC). The characterization of viral replication and inflammation profile, as well as the modulation of endothelial activation markers, were performed after 2 days in SARS-CoV-2 infected or not infected HAE-EC co-culture (Figure 1).

### 3.2. SARS-CoV-2 Infection of Human Airway Epithelial and Endothelial Cells

**Figure 2:**
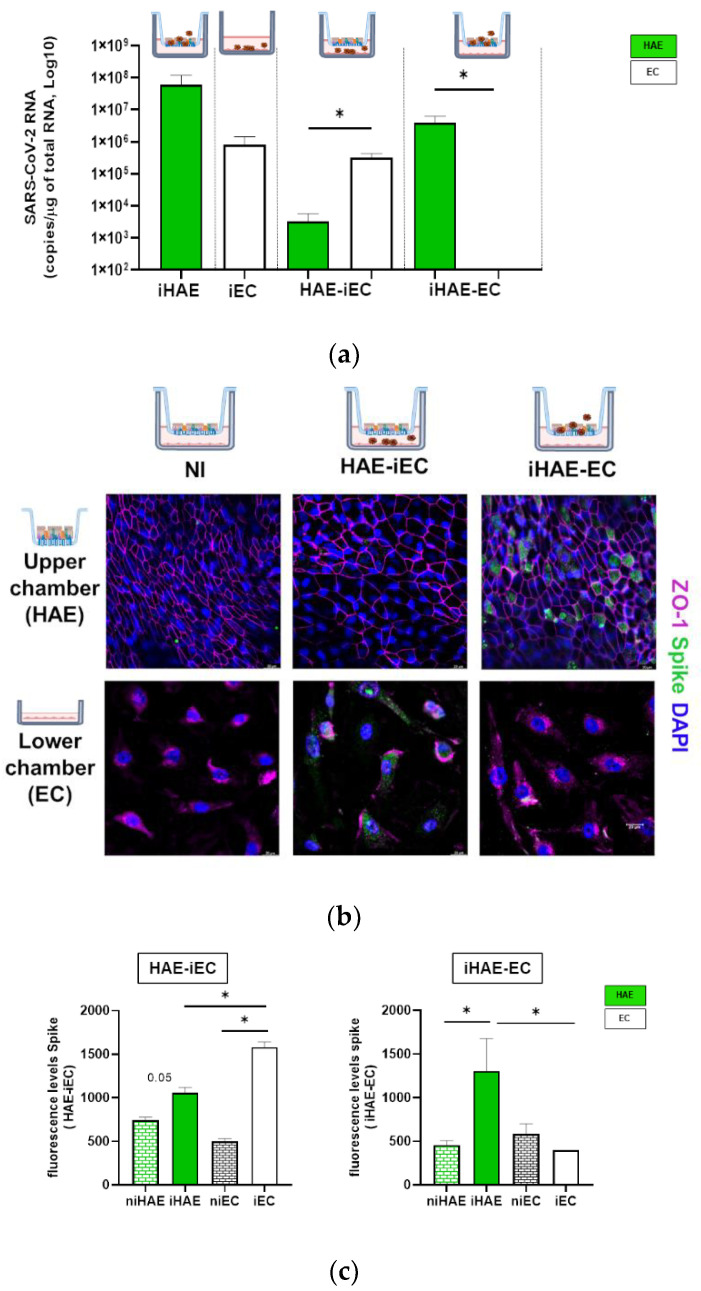
HAE-EC co-culture sustains SARS-CoV-2 replication. (**a**) SARS-CoV-2 RNA quantification in HAE (green bar) and ECs (white bar) at two days post-infection (n = 6 independent experiments) in iHAE, iEC cultured alone, or in co-culture HAE-iEC or iHAE-EC. Data are shown as mean + SEM. (**b**) Representative immunofluorescence analysis of upper and lower chamber after two days of co-culture with uninfected (NI) or SARS-CoV-2 infected EC (HAE-iEC) or HAE (iHAE-EC) (n = 6). Cells were stained for ZO-1 (purple) and Spike (green) antibodies. Thunder images were taken at a magnification of 63×. Nuclei were stained with DAPI (blue). Scale bars = 20 µm. (**c**) Fluorescence levels of Spike protein in HAE-EC co-culture in SARS-CoV-2 infected EC (HAE-iEC) or HAE (iHAE-EC). Values are presented as mean ± SEM. Mann-Whitney test was used for group comparison. * = *p* < 0.05.

The ability of HAE or ECs to sustain SARS-CoV-2 replication during a mono- or co-culture system was analyzed. SARS-CoV-2 replication was measured at two and six days post-infection comparing viral RNA levels associated with epithelial (iHAE) or endothelial cells (iEC). The level of SARS-CoV-2-RNA was higher in iHAE than in iECs monoculture (Figure 2a). Moreover, a low amount of SARS-CoV-2 RNA was also detected in HAE when cultured with infected-EC (HAE-iEC), suggesting that in co-culture the virus can go through the transwell and infect the HAE in the upper chamber (Figure 2a). In contrast, SARS-CoV-2 RNA in EC when co-cultured with infected HAE was not detected. Of note, SARS-CoV-2-RNA increased over time in HAE [23] but not in EC, suggesting that EC can be infected but they do not sustain effective viral replication (Supplementary Materials, Figure S1).

The presence of SARS-CoV-2 in ECs or in HAE during HAE-EC co-culture was also verified by immunofluorescence for the viral Spike protein and ZO-1 (Zonula occludens (ZO)-1 the tight junction protein expressed in all epithelial and endothelial cells [24]. The images and the quantitative analysis of the mean fluorescence Spike protein confirmed data of viral RNA (Figure 2b,c). Therefore, these data indicated that upon SARS-CoV-2 infection of HAE-EC co-culture, viral RNA and Spike protein of SARS-CoV-2 were detected both in ECs and in HAE, although effective viral replication was observed only in HAE.

### 3.3. Viral Replication in HAE Induces IFN-β Release and Modulation of p21 Expression in EC

**Figure 3:**
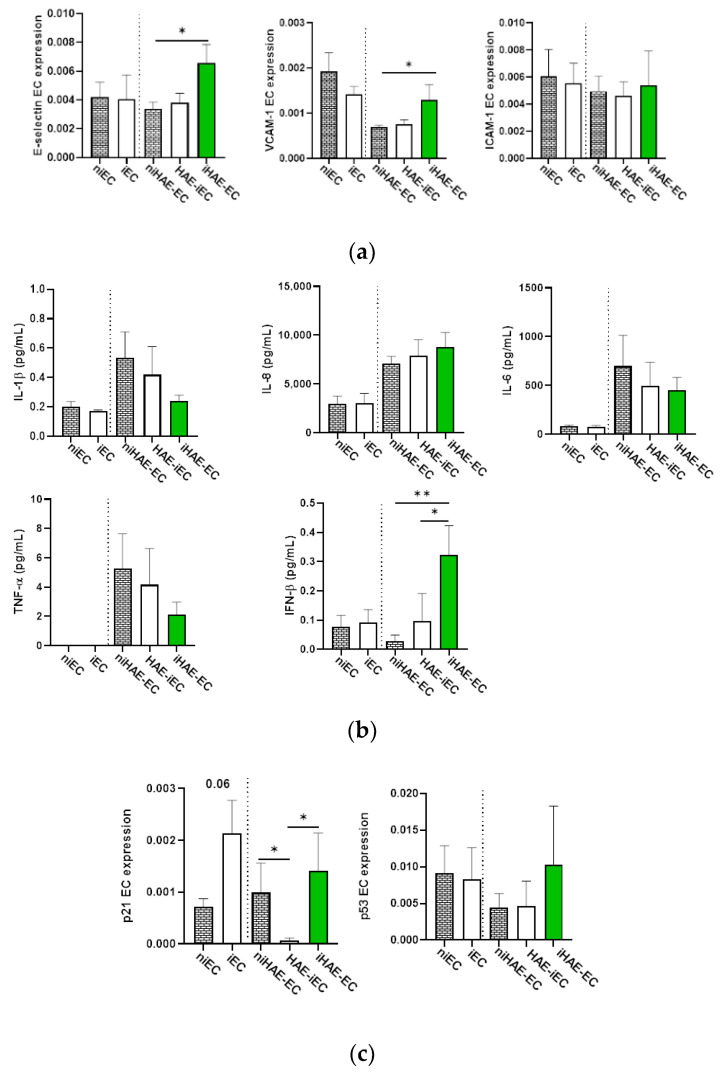
SARS-CoV-2 Viral replication in HAE induces IFN-β release and modulation of p21 expression in ECs. ECs gene expression analysis of adhesion molecules, senescence markers, and quantification of inflammatory cytokines and IFN-β levels. Data are presented in three different culture conditions (n = 6): (i) EC cultured alone, (ii) infected EC co-cultured with HAE (HAE-iEC) and (iii) infected HAE co-cultured with EC (iHAE-EC). As a control, uninfected EC cultured alone or in co-culture with HAE (nEC and nHAE-EC) are reported. (**a**) ECs gene expression analysis of E-selectin, ICAM-1, and VCAM-1; (**b**) Quantification of inflammatory cytokines (IL-1β, IL-6, IL-8) and IFN-β in the supernatant of indicated cultures; (**c**) ECs gene expression analysis of p21 and p53. Values are presented as mean ± SEM. Mann-Whitney test was used for group comparison. * = *p* < 0.05; ** = *p* < 0.01.

SARS-CoV-2 infection of EC did not induce an increase in the adhesion molecule expression. In contrast, the infection of HAE drove a significant increase of the adhesion molecules E-selectin and VCAM-1 expression in ECs (Figure 3a). To assess whether the inflammation was involved in the induction of adhesion molecules expression in ECs, we quantified the inflammatory cytokines IL-8, IL-1β, IL-6, and IFN-β in the supernatants of the co-cultures. No significant induction of inflammatory mediators was detected after infection, while an increase of IFN-β was observed after HAE (EC-iHAE) but not after EC infection (HAE-iEC) (Figure 3b). Moreover, we found that ISG15 expression was upregulated in ECs when co-cultured with infected HAE [fold increase of ISG15 expression (infected/uninfected) in iEC-HAE, median 0.8 vs. EC-iHAE, median 2.7], indicating the modulation of IFN-β signaling during co-culture with infected epithelium. Of note, the expression of IFN-β in ECs positively correlated with ICAM-1 and VCAM-1 expression (Figure S2).

It was previously demonstrated that senescent cells up-regulate the expression of the adhesion molecules E-selectin and ICAM-1 [25,26]. Therefore, to determine whether the induction of endothelial activation/damage genes in infected co-cultures could mirror senescence processes, we analyzed the expression of p21 and p53 in ECs [10]. SARS-CoV-2 infection of EC cultured alone induced a slight increase of p21 (Figure 3c, left panel). Unexpectedly, the presence of HAE was able to subvert this effect, inducing a significant decrease in p21 expression in EC. No differences were observed for p53 expression (Figure 3c, right panel).

### 3.4. Viral Replication in HAE Correlates with EC Activation, IFN-β Release, and p21 Expression

**Figure 4:**
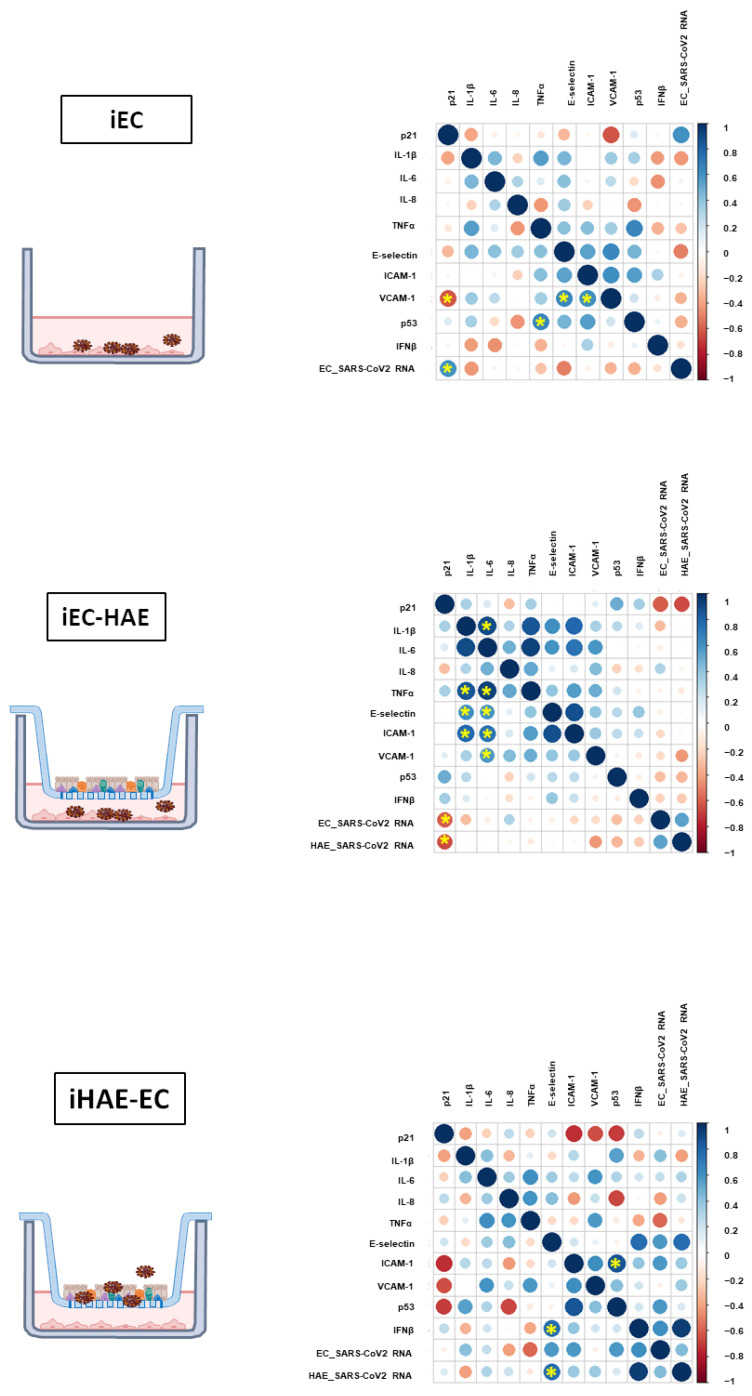
Associations among the inflammatory mediators, IFN-β, SARS-CoV-2 levels, adhesion molecules, and senescence markers expression in ECs cultured alone or with HAE. Spearman correlation matrices among IL-8, IL-1β, IL-6, and IFN-β levels, ECs E-selectin, ICAM-1, VCAM-1, p21/p53 expression, and SARS-CoV-2 RNA in ECs cultured alone (iEC, **upper** panel), SARS-CoV-2 infected ECs cultured with HAE (HAE-iEC, **middle** panel) and SARS-CoV-2 infected HAE cultured with EC (iHAE-EC, **lower** panel). The significant correlations (<0.05) are indicated with an asterisk.

In order to define the parameters affecting the EC activation, multiple correlation analysis (viral titer, adhesion molecules, cytokines, and senescent markers) was performed in the three experimental conditions: iEC, iEC-HAE, and iHAE-EC (Figure 4).

The correlations between EC activation and the other parameters have been observed mainly after HAE infection (iHAE-EC), confirming a bystander effect of HAE-derived signals on EC. Specifically: (i) viral RNA in HAE directly correlated with E-selectin; (ii) viral RNA in HAE directly induced IFN-β that, in turn, directly correlated with E-selectin; (iii) p53 expression correlated with ICAM-1. Of note, the linear regression analysis showed a significant result between p53 and ICAM-1 (r^2^: 0.5, *p* < 0.001), p53 and E-Selectin (r^2^: 0.4, *p* < 0.0002), while no significant regression was observed between viral RNA and all other parameters.

No correlations between viral replication and inflammatory cytokines have been reported. Nevertheless, a direct correlation between inflammatory cytokines on EC activation has been observed only when EC were infected in the presence of HAE (iEC-HAE).

Finally, a different relationship between viral RNA and p21 has been shown. Specifically, in EC mono-culture, the viral replication directly correlated with p21, suggesting the direct induction of senescence. Differently, the presence of HAE was able to subvert this effect, inducing a p21 decrease that was inversely correlated with viral RNA.

### 3.5. Impact of IFN-β on EC Senescence

**Figure 5:**
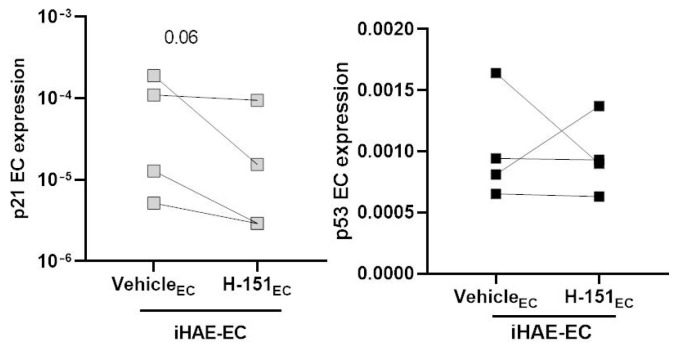
p21 and p53 ECs gene expression after H151 treatment in iHAE-EC co-culture. Gene expression analysis of p21 (**left** panel) and p53 (**right** panel) in ECs during co-culture with SARS-CoV-2 infected HAE (iHAE-EC) in the presence of H-151 (or vehicle) as indicated (n = 4). Values are presented as mean ± SEM. The Wilcoxon matched-pairs signed rank test was used for group comparison. *p*-value is reported in the graph.

IFN-β represents an important factor associated with impaired endothelial function and with antiviral and antiproliferative effects [27,28]. To better elucidate IFN-β contribution in EC senescence/activation, we inhibited IFN type I production by Stimulator of interferon genes (STING) inhibitor H-151 during co-culture with SARS-CoV-2 infected HAE. We, therefore, added H-151 directly on ECs (H-151_EC_) during infected co-culture. The addition in the basal chamber of H-151 induced a decrease in IFN-β release as shown in Figure S3. The inhibition of IFN-β resulted in a slight decrease in p21 expression in ECs (Figure 5). In contrast, H-151 did not modify the ECs activation markers as well as p53 expression in iHAE-EC.

Altogether, these results suggest that active SARS-CoV-2 replication in airway epithelium triggers the activation of pulmonary endothelial cells inducing an increase of IFN-β responses, which might contribute to accelerating the senescence process that may affect the effective virus replication.

## 4. Discussion

In the present study, we set up an organotypic model to dissect the crosstalk between airway epithelium and pulmonary endothelial cells during SARS-CoV-2 infection. This experimental system allowed us to distinguish the direct impact of SARS-CoV-2 infection on ECs activation from those induced by infected-HAE.

Other existing alveolar models are available [29]. Nevertheless, our experimental system allowed dissecting the interplay between epithelial and endothelial cells and the role of SARS-CoV-2 in modulating their cross-talk. Although some limitations of this system (e.g., cells were separated by a transwell insert, the organotypic model does not include immune cells), our results can be useful to better define the pathogenetic mechanisms occurring in the infected lung.

The ability of endothelial cells to sustain viral infection/replication is still controversial [29,30,31]. In our experimental model, we showed that human pulmonary endothelial cells are susceptible to SARS-CoV-2 infection but failed to sustain an active viral replication. SARS-CoV-2 direct infection of EC induced a slight increase of p21 but was not able to induce EC activation. Interestingly, when using the co-culture system, we demonstrated that SARS-CoV-2 infected-HAE is able to trigger endothelial adhesion molecule overexpression and IFN-β release, suggesting a bystander effect of dangerous soluble signals released by infected epithelium. Accordingly, endothelial activation was associated with viral replication in airway epithelium.

Recent evidence suggested that endothelial injury characterizing COVID-19 could be mediated by inflammatory factors released from infected cells such as epithelial cells rather than by direct viral infection [29]. Wang and co-authors [30] reported that while human pulmonary alveolar epithelial cells (HPAEpiC) effectively sustain viral replication, human lung microvascular endothelial cells were almost insusceptible to SARS-CoV-2 infection; however, these cells displayed mitochondrial fragmentation and Golgi apparatus alterations after treatment with the culture supernatant of SARS-CoV-2-infected HPAEpiC, indicating the main involvement of epithelium in EC damage. Our work confirms these data and adds the involvement of IFN-β signaling as a mechanism of endothelial damage triggered by epithelial signals.

Although a small number of studies are still available, several pieces of evidence suggest that the activation of senescence processes upon infections can play both beneficial (antiviral) and detrimental (tissue injury) roles. Viral infection is able to upregulate various senescence markers, including SA-b-gal, p16, p21, and pro-inflammatory senescence-associated secretory phenotypes (SASP) molecules IL-6 and IL-8 [32,33,34,35,36]. In a recent study, Lee et al. demonstrated that virus-induced senescence may represent a pathogenic trigger of COVID-19-related organ damage [37]. Indeed, in COVID-19 patients, an increase in senescence markers in their airway mucosa and an increase in serum levels of SASP factors have been described. In this work, the SARS-CoV-2 infection of EC induced an increase in p21 expression that may promote growth arrest, thus contributing to the establishment of an antiviral profile associated with the lack of effective viral replication in EC. Moreover, when iEC were co-cultured with HAE, p21 was reduced but its expression inversely correlated with viral RNA, confirming a dangerous signal released by epithelial cells in modulating viral replication in iEC. Interestingly, infected HAE, able to effectively sustain viral replication, induced IFN-β release and EC activation that, in turn, correlated with p53, suggesting the main role of infected HAE in inducing EC damage.

As shown in the primary murine fibroblast model and by chemotherapy tumor cells, viral-induced IFN-β release can be associated with the induction of cell senescence [18,38]. Accordingly, a strict relationship between senescence and STING signaling has been extensively shown [39]. Interestingly, in iHAE/EC co-culture, the block of IFN-β signaling pathways by a STING inhibitor, induced a slight decrease of p21, suggesting a possible role of viral-induced IFNs in modulating EC cell cycle and senescence. The inhibition of STING-failed to reduce ECs activation that maybe needs longer time or involve different intracellular pathways. Recently, STING has been proposed as a signal triggering pulmonary endothelial damage in vivo during SARS-CoV-2 infection [39]. Indeed, the inhibition of STING was able to improve the disease outcome after SARS-CoV-2 infection, highlighting the rationale for pathological type I IFN response in COVID-19 [40].

## 5. Conclusions

**Figure 6:**
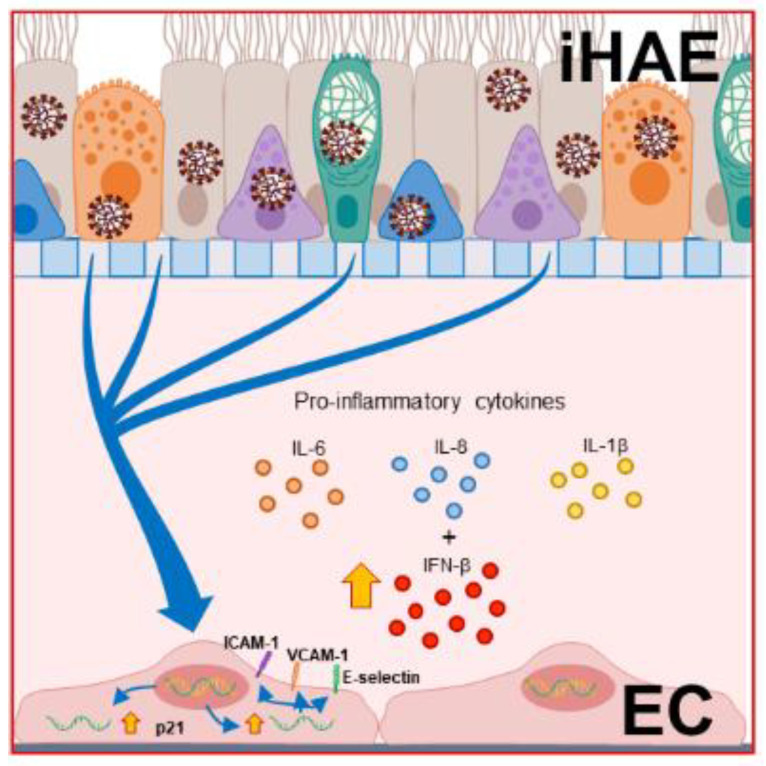
Proposed model of crosstalk between SARS-CoV-2 infected Human Airway Epithelium (iHAE) and Human Pulmonary Microvascular Endothelial Cells (ECs).

In conclusion, we describe the interplay between infected airway epithelium and endothelial cells, highlighting the role of dangerous soluble signals from HAE in inducing the ECs damage. As illustrated in Figure 6, we hypothesized that effective SARS-CoV-2 replication in airway epithelium induces IFN-β release by ECs, which can contribute to enhancing their activation and senescence and contributing to counteracting viral replication.

## Data Availability

Data available in a publicly accessible repository [https://rawdata.inmi.it/, accessed on 1 September 2022].

## Supplementary Materials

The following supporting information can be downloaded at: https://www.mdpi.com/article/10.3390/cells11182912/s1. Figure S1: Replicative dynamics of SARS-CoV-2 in HAE (left panel) and ECs (right panel) in terms of cell-associated viral RNA at two and six days post infection (n=6 independent experiments); Figure S2: Correlation analysis between IFN-β expression and ICAM or VCAM-1 in ECs; Figure S3: IFN-β levels released during SARS-CoV-2 infection of HAE in co-culture with ECs (iHAE-Ec) after H-151 (or vehicle) addition to the basal chamber.
