# Supplementary material for: SARS-CoV-2 Infection of Airway Epithelium Triggers Pulmonary Endothelial Cell Activation and Senescence Associated with Type I IFN Production
Source: Cells. 2022 Sep 17;11(18):2912. doi: 10.3390/cells11182912 (PMC9496907; doi:10.3390/cells11182912)
Supplement: Supplementary file 1 [file cells-11-02912-s001.zip › cells-1852217-supplementary.pdf]

Supplementary Figure S1.

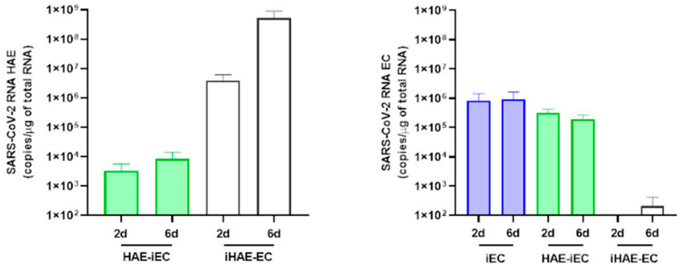

Replicative dynamics of SARS-CoV-2 in HAE (left panel) and ECs (right panel) in terms of cell-associated viral RNA at two and six days post infection (n=6 independent experiments). Data are shown as mean + SEM.

Supplementary Figure S2.

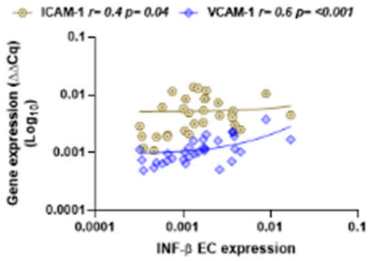

Correlation of IFN-β expression with ICAM and VCAM-1 in ECs; r and p are indicated in the graph.

Supplementary Figure S3.

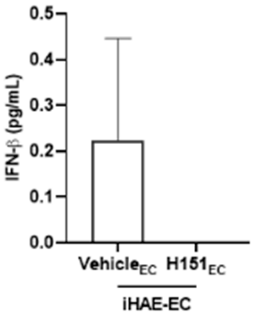

IFN-β levels released during SARS-CoV-2 infection of HAE in co-culture with ECs (iHAE-Ec) after H-151 (or vehicle) addition to the basal chamber. Values are presented as mean ± SEM.
